# Experimental Investigation of the Effects of a Blood Stopper Agent (Ankaferd Blood Stopper) on Bone Surfaces

**DOI:** 10.4274/Tjh.2012.0092

**Published:** 2013-06-05

**Authors:** Hasan Onur Şimşek, Mustafa Şenol Tüzüm, Timuçin Baykul, İnanç Elif Gürer, Cumhur İbrahim Başsorgun

**Affiliations:** 1 Süleyman Demirel University Faculty of Dentistry, Department of Oral and Maxillofacial Surgery, Isparta, Turkey; 2 Akdeniz University Faculty of Medicine, Department of Medical Pathology, Antalya, Turkey

**Keywords:** Hemostasis, Ankaferd Blood Stopper, Bone healing, Bone histomorphometry

## Abstract

**Objective:** This study aims to experimentally investigate the efficiency of Ankaferd Blood Stopper (ABS) on early and long-term bone healing and its effects on bone surfaces.

**Materials and Methods:** Thirty adult male Wistar albino rats were used in the study. These rats were randomly divided into three groups, and bilaterally bone defects were created in the femur of each rat. A 3.0-mm-deep monocortical circular defect was created with a 3.0 mm diameter trephine drill on the proximal part of the femur, and 0.05 mL ABS was applied to the experimental group while the control group was left untreated. Group 1, group 2, and group 3 rats were sacrificed on days 7, 28, and 42, respectively. Trabecular bone area (Tb.Ar), medullary bone diameter (Me.Dm), osteoblast area (Ob.Ar), osteoid area (O.Ar) and mineralized bone area (Md.Ar) were examined in the histomorphometric analysis. Also new bone formation was scored according to the histologic evaluation

**Results:** The results showed that while new the to day 7 experimental group showed much more bone formation than the to day 7 control group, there was no significant difference between the to day 28 and day 42 experimental groups and to day 28 and day 42 control groups. Accordingly, ABS applied in bone cavities only had a larger accelerator effect on bone healing for the seventh-day to day 7 experimental group. In clinical observations, no allergic or inflammatory reactions were observed on the skin and other preoperative and postoperative periods. Moreover in, the histomorphometric study, necrotic areas and infection areas were not observed.

**Conclusion:** ABS has an acceleratory effect on the short-term bone healing process and is a reliable agent for routine use. However, its effects on the long-term bone healing process are insignificant. We think that a wide series of research projects are required to confirm the effects of ABS speeding up the healing process in addition to its characteristic as a blood stopping agent.

**Conflict of interest:**None declared.

## Abstract (tr)

**Amaç:** Çalışmamızda deneysel olarak Ankaferd Blood Stopper (ABS)’ın erken ve geç dönem kemik iyileşmesi ve kemik yüzeylere etkisinin incelenmesi amaçlanmıştır.

**Gereç ve Yöntemler:** Çalışmaya 30 yetişkin erkek Wistar albino rat dahil edilmiştir. Denekler rastgele üç grubu ayrılmış ve kemik defektleri her bir femura uygulanmıştır. Kemik defektleri femurun proksimal kısmında 3,0 mm çapında trefin frez yardımıyla 3,0 mm derinliğinde, monokortikal ve dairesel olarak hazırlanmıştır. Deney grubuna 0,5 mL ABS uygulanırken kontrol grubuna herhangi bir madde uygulanmamıştır. Denekler 3 gruba ayrılarak sırasıyla yedinci, yirmi sekizinci ve kırk ikinci günlerde sakrifiye edilmiştir. Örneklerin histomorfometrik analizlerinde trabeküler kemik yüzey alanı, medullar kemik çapı, osteoblast yüzey alanı, osteoid kemik yüzey alanı ve mineralize kemik yüzey alanları incelenmiştir. Yeni kemik oluşumunun histolojik değerlendirmesi yapılarak, yeni kemik oluşum skorlaması yapılmıştır.

**Bulgular:** Çalışmamız sonucunda ABS kemik kavitelerinde uygulanmasının 7. gün deney grubunda kemik iyileşmesini hızlandırıcı etkileri üzerine daha net bulgulara ulaşılmış ve 7. gün sonuçlarına göre yeni kemik yapımını deney grubunda fazla olarak bulunmuştur. Ankaferd Blood Stopper kemik kavitelerinde uygulanmasının 28. ve 42. gün deney ve kontrol gruplarında benzer sonuçlara ulaşılmıştır. Klinik gözlemlerde intraoperatif ve postoperatif dönemde cilt ve diğer dokularda alerjik ve iltihabi reaksiyonlar gözlenmemiştir. Ayrıca histomorfometrik çalışmalarda nekrotik alanlara ve enfeksiyoz reaksiyonlara rastlanmamıştır.

**Sonuç:** Sonuç olarak, ABS kısa dönem kemik iyileşmesinde olumlu etkileri olabileceği gösterilirken, uzun dönem de kemik yapımını artırıcı etkileri olduğu gösterilmemiştir. Bununla birlikte ameliyat öncesi ve sonrasında herhangi bir yabancı cisim reaksiyonu veya nekrotik alanlara rastlanılmaması ve komplikasyon oluşturmaması pozitif etkileri olarak değerlendirilebilir. Ankaferd Blood Stopper’in kanama durdurucu ajan olmasının yanında kemik iyileşmesini hızlandırıcı etkileri, geniş serili araştırmalarla desteklenmesi gerektiğini düşünmekteyiz.

## INTRODUCTION

All surgical procedures in oral and maxillofacial surgery within the area of operation lead to varying degrees of bleeding. Providing an effective hemostasis agent during an operation is one of the most basic surgical principles. Bleeding control is vital for pathological hemostasis patient groups. Excessive bleeding in these patients is not only distressing for the patient, but also prevents the completion of procedures. Bleeding in patients can cause excessive blood loss, poor wound healing, or infection [1,2]. In addition to routine hemostatic methods, various local hemostatic agents are widely used for bleeding management [3,4,5,6]. Ankaferd Blood Stopper (ABS), which is manufactured for use in dentistry, is an effective hemostatic agent. ABS consists of 5 different plant extracts: Urtica dioica, Vitis vinifera, Glycyrrhiza glabra, Alpinia officinarum, and Thymus vulgaris (Table 1). Goker et al. suggested that ABS stimulated the formation of an encapsulated protein network that provides focal points for erythrocyte aggregation [7]. In addition, the combination of these plants in ABS appears to provide a unique composition for tissue oxygenation and the physiological hemostatic process without disturbing the levels of any individual clotting factor [7]. Various experimental and clinical studies have demonstrated the effect of ABS on bleeding control [8,9,10,11,12,13,14].

Many dentistry studies have reported the reliability and availability of ABS [15,16,17]. Besides its bleeding control feature, ABS reportedly accelerates early bone healing and formation [18]. The aim of our study was to investigate the efficiency of the early and long-term bone healing benefits of ABS and its clinical usage on bone surfaces.

## MATERIALS AND METHODS

**Animal Care and Experimental Procedure**

The study was accepted by Süleyman Demirel University’s Scientific Research Projects Unit. Süleyman Demirel University’s Animal Tests Local Ethics Council approved the treatment of animals in the study. Surgical procedures were performed at Süleyman Demirel University’s Experimental Animal Production and Experimental Research Laboratory.

A total of 30 adult male Wistar albino rats with an average weight of 340 g were used in the study. The animals were housed in groups of 5 per cage and fed standard pellets and water in a temperature-regulated room (22 °C, 55% humidity, and a 12-h light/dark cycle) without any limitation of mobilization. These rats were randomly divided into 3 groups [group 1 (n=10), group 2 (n=10), group 3 (n=10)] and bilateral bone defects were created in the femur of each rat. Right femoral defects served as the experimental group, while left femoral defects served as the control group (subgroup a, experimental group; subgroup b, control group) (Table 2). 

**Surgical Procedure**

**Figure 1:**
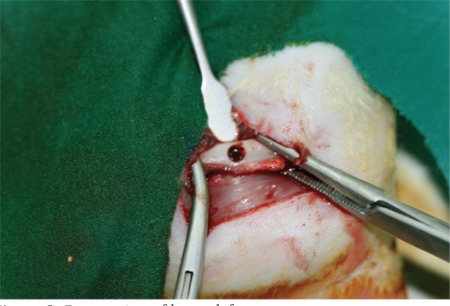
Preparation of bone defect.

The preoperative weights of all rats were measured, and general anesthesia was achieved by intramuscularly applying 15 mg/kg ketamine HCl (10% Alfamine®) and 60 mg/kg xylazine HCl (Alfazyne®, 2%) to each rat. After achieving an adequate depth of anesthesia, the femoral regions were scrubbed with 10% povidone-iodine solution (İsosol®, Central Lab., İstanbul). An approximately 2-cm longitudinal skin incision was made in the operation area. A full-thickness flap was prepared and the bone surface of the femur was exposed. Under saline solution irrigation, a monocortical circular defect 3.0 mm deep was created with a 3.0-mm-diameter trephine drill on the proximal part of the femur (Figure 1). The experimental group was treated with 0.5 mL of ABS while the control group was left untreated. Muscle and subcutaneous tissues were sutured with a 4/0 absorbable suture (Buril 4/0, AlfaTıp, Bursa), and the skin was sutured with a 3/0 silk suture (Bursilk® USP 3/0, Göksel Med., Bursa). After surgery, intramuscular amikacin sulfate antibiotics (Amikozit, Eczacıbaşı-Zenitiva®) were administered twice a day for 3 days postoperatively. Group 1, group 2, and group 3 rats were sacrificed on days 7, 28, and 42, respectively. During the operations, one of the rats (group 3) died due to a complication with the general anesthesia. 

**Histomorphometric Procedures**

Histomorphometric examinations were performed at Akdeniz University’s Department of Medical Pathology. Fifty-eight specimens were evaluated and fixed in a 10% neutral buffered formalin solution for at least 72 h. The specimens were placed into 10% silver nitrate for 24 h. A TBD-2 solution was used for the decalcification process. The specimens were cut transversally into semi-serial sections of 3-4 µm and stained with hematoxylin–eosin (HE) and Van Gieson solution.

**Figure 2:**
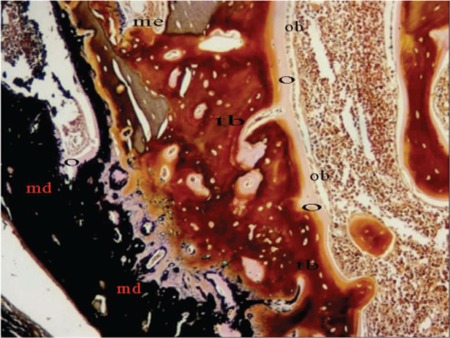
Histomorphometric section of group 3b (HE, 100x). O: osteoid, Ob: osteoblast, Tb: trabecular bone, Me: medullary bone, Md: mineralized bone.

**Figure 3:**
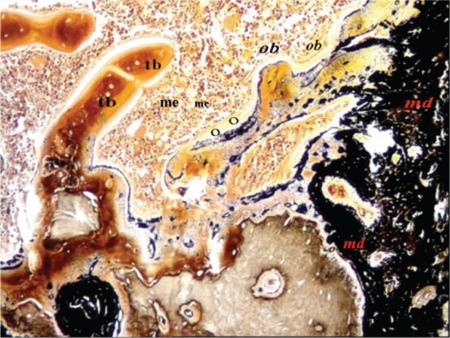
Histomorphometric section of group 3b (HE, 200x). O: Osteoid, Ob: osteoblast, Tb: trabecular bone, Me: medullary bone, Md: mineralized bone.

The specimens were analyzed using the AxioVision Release 4.7.1 software program. Histomorphometric parameters of medullary bone diameter, trabecular bone area, osteoid area, mineralized bone area, and osteoblast area were measured. New bone formation was scored according to histologic evaluation (Table 3). Parameters for histomorphometry were derived from the work of Parfitt et al. [19]. Medullary bone diameter and trabecular bone area at 40x (4/0.10 lens, 10/23 ocular) and osteoid area, mineralized bone area, and osteoblast area at 400x (40/0.65 lens, 10/23 ocular) were measured (Figures 2 and 3).

## RESULTS

**Statistical Analysis **

Statistical analysis was carried out using SPSS 10.0 (SPSS Inc., Chicago, IL, USA). The results are expressed as mean ± standard deviation. The Wilcoxon and chi-square tests were used to compare data between the control and experimental samples. Differences at p≤0.05 were considered significant. 

Groups 1 a and 1 b were compared according to medullary bone diameter (p=0.575), trabecular bone area (p=0.646), osteoid area (p=0.508), mineralized bone area (p=0.799), and osteoblast area (p=0.022) (Table 4). Although statistically significant differences (Wilcoxon test) were not obtained (excluded osteoblast area), mean values for the experimental group were higher than those for the control group. In terms of histological scoring of new bone tissue in the defect area, values were higher in the experimental group than in the control group and the chi-square test was statistically significant (p=0.013).

Groups 2a and 2b were compared according to medullary bone diameter (p=0.333), trabecular bone area (p=0.333), osteoid area (p=0.575), mineralized bone area (p=0.386), and osteoblast area (p=0.445) (Table 5). Although statistically significant differences (Wilcoxon test) were not obtained, medullar bone diameter, trabecular bone area, and osteoblast area mean values of the experimental group were higher than those of the control group. Osteoid area and mineralized bone area mean values were higher in the control group. In terms of histological scoring of the new bone tissue in defect areas, values were higher in the control group than in the experimental group and the chi-square test was not statistically significant (p=0.572).

Groups 3a and 3b were compared according to medullary bone diameter (p=0.214), trabecular bone area (p=0.441), osteoid area (p=0.314), mineralized bone area (p=0.314), and osteoblast area (p=0.374) (Table 6). Although statistically significant differences (Wilcoxon test) were not obtained, the medullary were 

not obtained, the medullar bone diameter and trabecular bone area of the experimental group were higher in mean value than in the control group. Osteoblast area, osteoid area, and mineralized bone area mean values were also higher than in the control group. In terms of histological scoring of new bone tissue in defect areas, values were higher in the experimental group than in the control group and the chi-square test was not statistically significant (p=0.368).

## DISCUSSION

ABS is a plant-based hemostatic agent composed of a standardized mixture of Urtica dioica, Vitis vinifera, Glycyrrhiza glabra, Alpinia officinarum, and Thymus vulgaris. All of these plants are individually effective on the endothelium, blood cells, angiogenesis, cellular proliferation, vascular dynamics, and mediators [7]. The basic mechanism of ABS ensures formation of focal points for erythrocyte aggregation through the formation of an encapsulated protein network. Reportedly, following ABS use, a decrease in plasma fibrinogen activity and a drop in fibrinogen antigen levels were observed and, accordingly, thrombin time was prolonged. Moreover, the total protein, albumin, and globulin levels in plasma were significantly reduced. Therefore, ABS affects fibrinogen-erythrocyte agglutination, leading to a protein network stimulating erythrocyte aggregation [7]. 

During study carried out in order to clarify ABS’s mechanism of action on coagulation by functional proteomics analysis, many plant and human proteins have been identified in ABS’s content. Demiralp et al. evaluated the study as enlightening in terms of the investigation of hemostatic, wound healing, and anti-inflammatory effects of ABS [20].

The hemostatic properties of ABS have been demonstrated by studies carried out in dentistry and other fields [8,9,10,11,12,13,14,15,16,17]. Baykul et al. reported at the end of a study performed on 4 patients with von Willebrand disease, chronic liver disease, and mitral valve replacement that ABS was an effective agent in stopping localized bleeding during tooth extraction with diathesis patients [15]. Ercetin et al.’s study on the reliability and qualification of ABS in dental surgery and bleeding showed that ABS can be beneficial for local hemostasis, wound healing, and infection control during periodontal surgery and tooth extraction [16]. Sonmez et al. reported that ABS use gave effective results in the early stage in a patient with type II Glanzmann thrombocytopenia [17].

In addition to its hemostatic agent properties, ABS has been demonstrated to cause rapid healing in bone tissue. In a study performed with Ankaferd Blood Stopper on 16 Wistar albino male rats, Işler et al. found that at the end of day 7, the presence of new bone formation in the ABS group was significantly higher than that in the control group, and they reported that ABS application had a positive impact on early-stage bone tissue healing [18]. The objective of our study was to experimentally study the early and late-stage effects of ABS on bone surfaces and its reliability for clinical applications. During our investigation, medullar bone diameter and trabecular surface area of the defect region, osteoblast surface area in the defect region, and mineralized bone surface area were histomorphometrically measured. Moreover, new bone formation and necrosis areas were examined. Foreign body reactions and infection findings were clinically investigated. 

The absence of any statistically significant difference between medullar bone area measurements in all 3 groups in our study’s findings suggests that section areas measured had similar values, and the study shows the reliability of the agent. Trabecular bone, which was another parameter of our study, is considered the fundamental structural component of cancellous bone [19]. The amount of trabecular bone provides histological information about new bone formation [21,22,23,24]. A study by Chiba et al. reported that hematoma started to change to fibrous tissue during a 7-day period and to conjunctive bone on day 10 [23]. Pereira et al. examined the osteoblasts in newly forming trabecular areas observed on the defect site and reported that the osteoblasts had a cubic structure at the end of day 7 and that immature bones formed on day 7 of the study [25]. For the assessment of new bone formation, osteoblast area, osteoid area, and the amount of mineralized bone are taken into consideration as other important parameters. An immature structure was observed in newly forming trabecular bone in the early bone healing stage in our study. High values of osteoblast, osteoid, and mineralized areas in the test group suggest a higher amount of new bone formation. These findings were supported by a high amount of conjunctive bones in the test group on day 7 and the statistically significant nature of these values. These results suggest that ABS may have positive effects on early-stage bone healing.

ABS is thought to create new stimulation in the area to which it is applied. In a previous study, potential transcription factor changes caused by ABS on human umbilical vein endothelial cells were investigated in order to study the effect of ABS on the endothelium. It was confirmed that ABS is extremely effective in stopping bleeding by its accelerated speed of complex formation between the cells, and it was concluded that the bond formed inside the complex is extremely strong. It was stated that at low doses, it is effective both outside and inside the cells, and it can affect many mechanisms inside the cell [26].

In late-stage findings of our study, the control group histomorphometrically showed superiority in osteoblast and osteoid surface areas and accordingly in mineralized surface areas; however, superiority of new bone formation sites could not be shown. Failure of either the study or control group to show superiority can be ascribed to the ongoing normal ossification process. Moreover, the regular increase shown in trabecular bone and mineralized bone surface areas between the groups according to study days 7, 28, and 42 may suggest that ABS has no negative or delaying effects on physiological bone healing. Although ABS has boosting effects on bone healing during the early period, it is possible that these effects are eliminated with time during the normal physiological bone healing process.

Reactions in tissues caused by the substances used to stop bleeding are important for the reliability of the materials used. Absence of any necrosis areas and foreign body reactions in tissues at the end of the administration indicates the reliability of the substance. Hemostatic agents administered on ranges of tension come in contact with bone surfaces. In a study by Bilgili et al., high-dosage systemic administration of ABS in an in vivo animal model was shown to not cause mucosal toxicity, hematotoxicity, hepatotoxicity, nephrotoxicity, or biochemical toxicity [27]. In another study in which the in vitro antimicrobial activity of ABS was investigated, clinical isolate obtained by the agar well diffusion technique from 102 patients was examined. Fifteen different microorganisms were assessed during the study. It was found that all gram-negative and gram-positive microorganisms had zones against ABS with a diameter of 15 mm on average and in the range 10-18 mm, and ABS was reported to be antimicrobially active [28]. Hence, reactions caused by ABS in tissue were also investigated in our study. The findings showed that ABS use did not cause any foreign body reaction, and no necrotic areas were formed. No swelling, inflammatory reaction, or allergic reactions were observed clinically in the surgery sites. The absence of any foreign body reaction and only one necrotic area are supported by the findings of Işler et al. [18]. No necrotic tissue was observed in bone tissue and defect areas during the histomorphometric examination in our study. These findings support each other.

As a conclusion of this study, ABS applied in bone cavities was found to have an accelerator effect on short-term bone healing, but no effect on long-term bone healing. Even though its superiority in terms of physiological healing during the long-term bone healing process could not be shown, we think that the speeding up of short-term healing is significant in the recovery of function, phonetics, and aesthetics of patients. In addition, ABS showed positive features in that it did not cause any foreign body reaction or necrotic areas, and no complications during clinical observations before and after surgery were observed. We think that a wide series of research projects are required to confirm the effects of ABS in speeding up the healing process in addition to its characteristics as a blood-stopping agent. 

## CONFLICT OF INTEREST STATEMENT

The authors of this paper have no conflicts of interest, including specific financial interests, relationships, and/or affiliations relevant to the subject matter or materials included.

## Figures and Tables

**Table 1:**
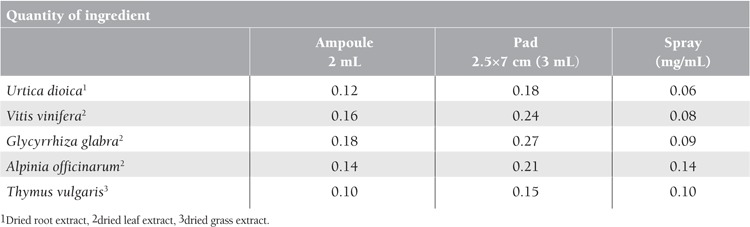
Ingredients in the ampoule, spray, and pad forms of ABS.

**Table 2:**
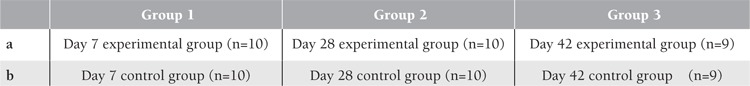
Classification of groups.

**Table 3:**
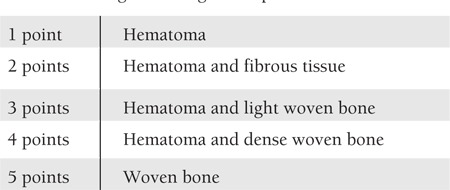
Histological scoring of the specimens.

**Table 4:**
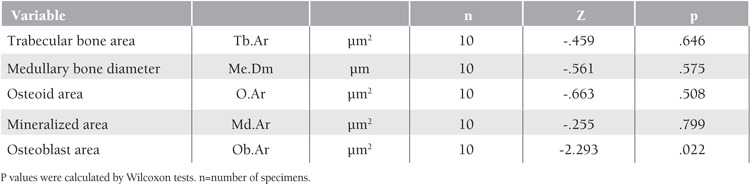
Statistical analysis of groups 1a and 1b.

**Table 5:**
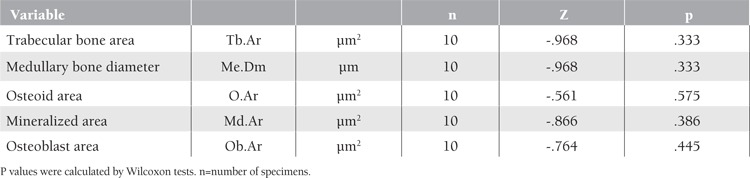
Statistical analysis of groups 2a and 2b.

**Table 6:**
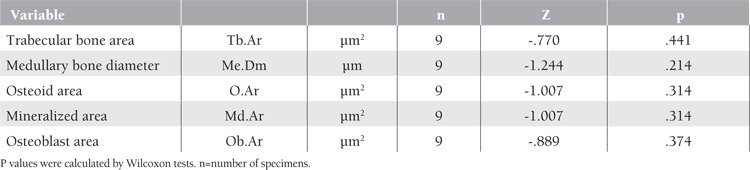
Statistical analysis of groups 3a and 3b.
